# Vaccine Clinical Trials: Focus on Cardiovascular Disease

**DOI:** 10.1016/j.jacadv.2026.102994

**Published:** 2026-07-22

**Authors:** Orly Vardeny

**Affiliations:** University of Minnesota, Minneapolis, Minnesota, USA

**Keywords:** cardioprotection, cardiovascular disease, influenza, vaccine

**Figure:**
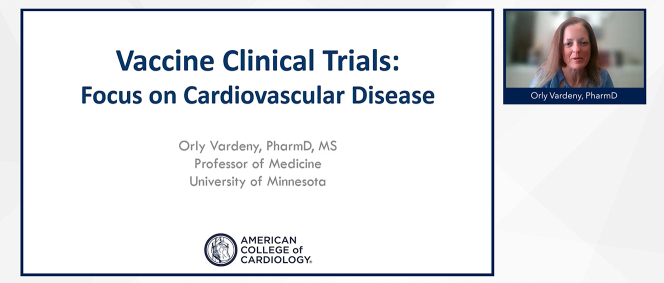
The following is the video related to this paper.

Influenza leads to significant morbidity and mortality each year. Respiratory symptoms are just a small part of influenza’s overall impact, as the virus is linked to a wide range of systemic health issues. Viral illness can be destabilizing for individuals with chronic medical conditions, including those with underlying cardiovascular disease. Infection has been temporally associated with acute cardiac events such as myocardial infarction and can precipitate acute decompensated heart failure.^1^^,^^2^

Influenza vaccination as a strategy to reduce the risk for adverse cardiovascular sequelae has been well studied. The effect of influenza vaccination on reducing the risk for acute cardiac events was shown in a meta-analysis of smaller randomized controlled trials.^3^ Receipt of influenza vaccine, compared with placebo or no vaccine, was associated with a 36% reduced risk for major adverse cardiovascular events, most notably among those with more recent acute coronary syndrome. examining receipt of influenza vaccine vs either placebo or control.

Earlier randomized trials indicated cardiac benefits of vaccination. The IAMI (Influenza Vaccination After Myocardial Infarction) trial randomized 2,571 patients with acute coronary syndrome or those undergoing angiography to receive a vaccine or placebo.^4^ It examined the primary composite outcome of all-cause death, myocardial infarction, or stent thrombosis within 12 months’ postvaccination. The trial was stopped early prior to completing enrollment due to the COVID-19 pandemic; nonetheless, there was a 28% reduced risk of the primary endpoint with the influenza vaccine compared with placebo.

In the IVVE (Influenza Vaccine to Prevent Adverse Vascular Events) study, 5,129 patients with symptomatic heart failure were randomized to receive an influenza vaccine vs placebo.^5^ No significant differences were found between treatment groups for the primary cardiovascular disease composite endpoints. However, there appeared to be a benefit with influenza vaccination on the primary endpoint when events were restricted to those that occurred during influenza season, during which the benefit of the vaccine is biologically plausible.

The composition of influenza vaccines changes annually, as determined by the World Health Organization, to reflect widely circulating virus strains. During the 2025 to 2026 season, the influenza vaccine contained 3 virus antigens, 2 from the A/lineage and 1 from the B/lineage. Efficacy of influenza vaccines varies from season to season, contingent on the match between vaccine antigens and circulating virus strains, the virulence of the predominant viral strain, and patient-specific factors such as age and comorbidities that can influence the magnitude of immune response to the vaccine. Older adults exhibit immunosenescence (age-related decline in immune function), which increases their risk for infection and leads to decreased immune responses to vaccines. The Advisory Committee on Immunization Practices currently recommends the use of an enhanced influenza vaccine formulation for older adults, such as the high-dose egg-based inactivated influenza vaccine, an adjuvanted vaccine, or a recombinant influenza vaccine.^6^ A prior study reported reduced humoral immune responses to influenza vaccine antigens in patients with heart failure compared with age-matched control subjects.^7^ In a subsequent small, randomized study, the use of double-dose inactivated influenza vaccine resulted in higher antibody titers to all 3 vaccine antigens compared with standard-dose vaccine.^8^

A few recent trials have evaluated different influenza vaccine dose formulations with respect to cardiac protection. INVESTED (Influenza Vaccine to Effectively Stop Cardio Thoracic Events and Decompensated Heart Failure) studied 5,260 individuals (mean age 65.5 years) in the United States and Canada with high-risk cardiovascular disease (within 12 months of acute myocardial infarction or within 24 months of hospitalization for heart failure, and an additional risk factor).^9^ The investigators found no significant difference between high-dose and standard-dose influenza vaccine strategies on the time to first all-cause death or cardiopulmonary hospitalization across 3 influenza seasons (HR: 1.06 [95% CI: 0.97-1.17]; *P* = 0.21). In Brazil, the VIP-ACS (Vaccination Against Influenza to Prevent Cardiovascular Events After Acute Coronary Syndromes) pragmatic trial similarly showed no benefit of double-dose over standard-dose influenza vaccine among 1,801 patients hospitalized with acute coronary syndrome across 2 influenza seasons.^10^ The hierarchical composite primary outcome encompassing all-cause death, myocardial infarction, stroke, unstable angina, heart failure hospitalization, urgent coronary revascularization, and respiratory hospitalization was comparable between groups (win ratio: 1.02 [95% CI: 0.79-1.32]; *P* = 0.84).

## Large-Scale randomized trials of high-dose vaccine

In Denmark, the DANFLU-1 (Feasibility of Randomizing Danish Citizens Aged 65-79 Years to High-Dose Quadrivalent Influenza Vaccine vs Standard-Dose Quadrivalent Influenza Vaccine in a Pragmatic Registry-Based Setting) trial enrolled 12,477 older adults (mean age: 71.7 years) in a pragmatic registry-based comparison of high-dose vs standard-dose quadrivalent influenza vaccine.^11^ It found no significant difference in relative vaccine effectiveness (rVE) for cardiorespiratory (rVE: 12.1% [95% CI: −15.5% to 33.3%]) or cardiovascular (rVE: −1.0% [95% CI: −39.1% to 26.6%]) outcomes. High-dose vaccine was associated with significant reductions in influenza or pneumonia hospitalization (rVE: 64.4% [95% CI: 24.4%-84.6%]) and all-cause mortality (rVE: 48.9% [95% CI: 11.5%-71.3%]), driven primarily by the respiratory hospitalization component; the study was not powered for clinical outcomes, however.

The larger DANFLU-2 (A Pragmatic Randomized Trial to Evaluate the Effectiveness of High-Dose Quadrivalent Influenza Vaccine vs Standard-Dose Quadrivalent Influenza Vaccine in Older Adults) trial was completed in Denmark over 3 consecutive influenza seasons (2022-2023, 2023-2024, and 2024-2025).^12^ A total of 332,438 participants were randomized to treatment (166,218 to high-dose inactivated influenza vaccine and 166,220 to standard-dose inactivated influenza vaccine); their mean age was 73.7 years, and 27.4% had a history of cardiovascular disease. The primary endpoint of hospitalization for influenza or pneumonia was not significantly different between vaccine groups (rVE: 5.9% [95% CI: −2.1% to 13.4%]; *P* = 0.14). A secondary analysis suggested a lower incidence of cardiorespiratory hospitalizations in recipients of high-dose vaccine (rVE: 5.7% [95.2% CI: 1.4%-9.9%]), although the results need to be interpreted cautiously given the neutral results for the primary endpoint. Additional prespecified and exploratory secondary analyses further identified a directional benefit of high-dose vaccine on cardiovascular and cardiorespiratory hospitalizations, with the most pronounced signal observed for heart failure hospitalization (rVE: 19.5% [95% CI: 3.3%-33.1%]).

Another large, pragmatic randomized controlled trial named GALFLU (Pragmatic Randomized Trial to Evaluate the Effectiveness of High-Dose Quadrivalent Influenza Vaccine vs Standard-Dose Quadrivalent Influenza Vaccine in Adults Aged 65-79 Years in Galicia, Spain) was conducted in Spain during the 2023 to 2024 and 2024 to 2025 seasons among 103,169 individuals aged 65 to 79 years (mean age 72.3 years; 12.5% with a history of cardiovascular disease) who were allocated to high-dose vs standard-dose influenza vaccine.^13^ The primary endpoint was hospitalization for influenza or pneumonia, which occurred in 0.26% in the high-dose group compared with 0.34% of standard-dose recipients (relative risk: 0.76; relative vaccine effectiveness: 23.7% [95% CI: 6.6%-37.7%]). Due to a lower-than-expected event rate, descriptive statistics were used to report trial results. The GALFLU trial was designed a priori to align methodologically with the DANFLU-S trial, called the FLUNITY-HD study (Pooled Analysis of Methodologically Harmonized Pragmatic Randomized Trials of High-Dose vs. Standard-Dose Influenza Vaccine Against Severe Clinical Outcomes). In one FLUNITY-HD analysis that encompassed 466,320 participants from both trials, the risk for hospitalization for influenza and pneumonia was lower in the high-dose influenza vaccine group (0.56%) compared with the standard-dose vaccine group (0.62%; rVE: 8.8% [95% CI: 1.7%-15.5%]; 1-sided *P* = 0.0082).^14^

In summary, influenza vaccination represents a well-established and underutilized cardiovascular prevention strategy. The totality of evidence supports universal influenza vaccination in patients with cardiovascular disease and warrants continued investigation into the optimal vaccine formulation for high-risk subgroups. This includes older adults, those with heart failure, and individuals with impaired immune responses, in whom standard-dose strategies may be insufficient and enhanced formulations (including high-dose vaccines) may confer incremental benefit.

## Funding support and author disclosures

Dr Vardeny has received research support to her institution from 10.13039/100004325AstraZeneca, 10.13039/100004326Bayer, CPC Clinical Research, and Cardurion; and consulting fees from 10.13039/100004325AstraZeneca, 10.13039/100004326Bayer, 10.13039/100014941Cytokinetics, 10.13039/501100004191Novo Nordisk, 10.13039/100019533Moderna, and 10.13039/100004319Pfizer.

## Appendix

Video 1 Vaccine Clinical Trials With Emphasis on Patients With Cardiovascular Disease. The video explores how influenza can affect patients with cardiovascular disease and how vaccinations can help reduce risks.
